# Predictive modeling of significance thresholding in activation likelihood estimation meta-analysis

**DOI:** 10.1162/imag_a_00423

**Published:** 2025-01-10

**Authors:** Lennart Frahm, Kaustubh R. Patil, Theodore D. Satterthwaite, Peter T. Fox, Simon B. Eickhoff, Robert Langner

**Affiliations:** Department of Psychiatry, Psychotherapy and Psychosomatics, School of Medicine, RWTH Aachen University, Aachen, Germany; Institute of Neuroscience and Medicine (INM7: Brain and Behavior), Research Centre Jülich, Jülich, Germany; Institute of Systems Neuroscience, Medical Faculty, Heinrich Heine University, Düsseldorf, Germany; Department of Psychiatry, Perelman School of Medicine, University of Pennsylvania, Philadelphia, PA, United States; Penn Lifespan Informatics and Neuroimaging Center, Perelman School of Medicine, University of Pennsylvania, Philadelphia, PA, United States; Research Imaging Institute, University of Texas Health Science Center, San Antonio, TX, United States; Departments of Radiology, Neurology, Psychiatry and Behavioral Sciences, and Physiology, University of Texas Health Science Center, San Antonio, TX, United States

**Keywords:** activation likelihood estimation, Monte-Carlo simulation, multiple comparison correction, TFCE, cFWE, XGBoost

## Abstract

Activation Likelihood Estimation (ALE) employs voxel- or cluster-level family-wise error (vFWE or cFWE) correction or threshold-free cluster enhancement (TFCE) to counter false positives due to multiple comparisons. These corrections utilize Monte-Carlo simulations to approximate a null distribution of spatial convergence, which allows for the determination of a corrected significance threshold. The simulations may take many hours depending on the dataset and the hardware used to run the computations. In this study, we aimed to replace the time-consuming Monte-Carlo simulation procedure with an instantaneous machine-learning prediction based on features of the meta-analysis dataset. These features were created from the number of experiments in the dataset, the number of subjects per experiment, and the number of foci reported per experiment. We simulated 68,100 training datasets, containing between 10 and 150 experiments and computed the vFWE, cFWE, and TFCE significance thresholds. We then used this data to train one XGBoost regression model for each thresholding technique. Lastly, we validated the performance of the three models using 11 independent real-life datasets (21 contrasts) from previously published ALE meta-analyses. The vFWE model reached near-perfect prediction levels (R² = 0.996), while the TFCE and cFWE models achieved very good prediction accuracies of R² = 0.951 and R² = 0.938, respectively. This means that, on average, the difference between predicted and standard (monte-carlo based) cFWE thresholds was less than two voxels. Given that our model predicts significance thresholds in ALE meta-analyses with very high accuracy, we advocate our efficient prediction approach as a replacement for the currently used Monte-Carlo simulations in future ALE analyses. This will save hours of computation time and reduce energy consumption. Furthermore, the reduced compute time allows for easier implementation of multi-analysis set-ups like leave-one-out sensitivity analysis or subsampling.

## 1 Introduction

**Fig. 1.**
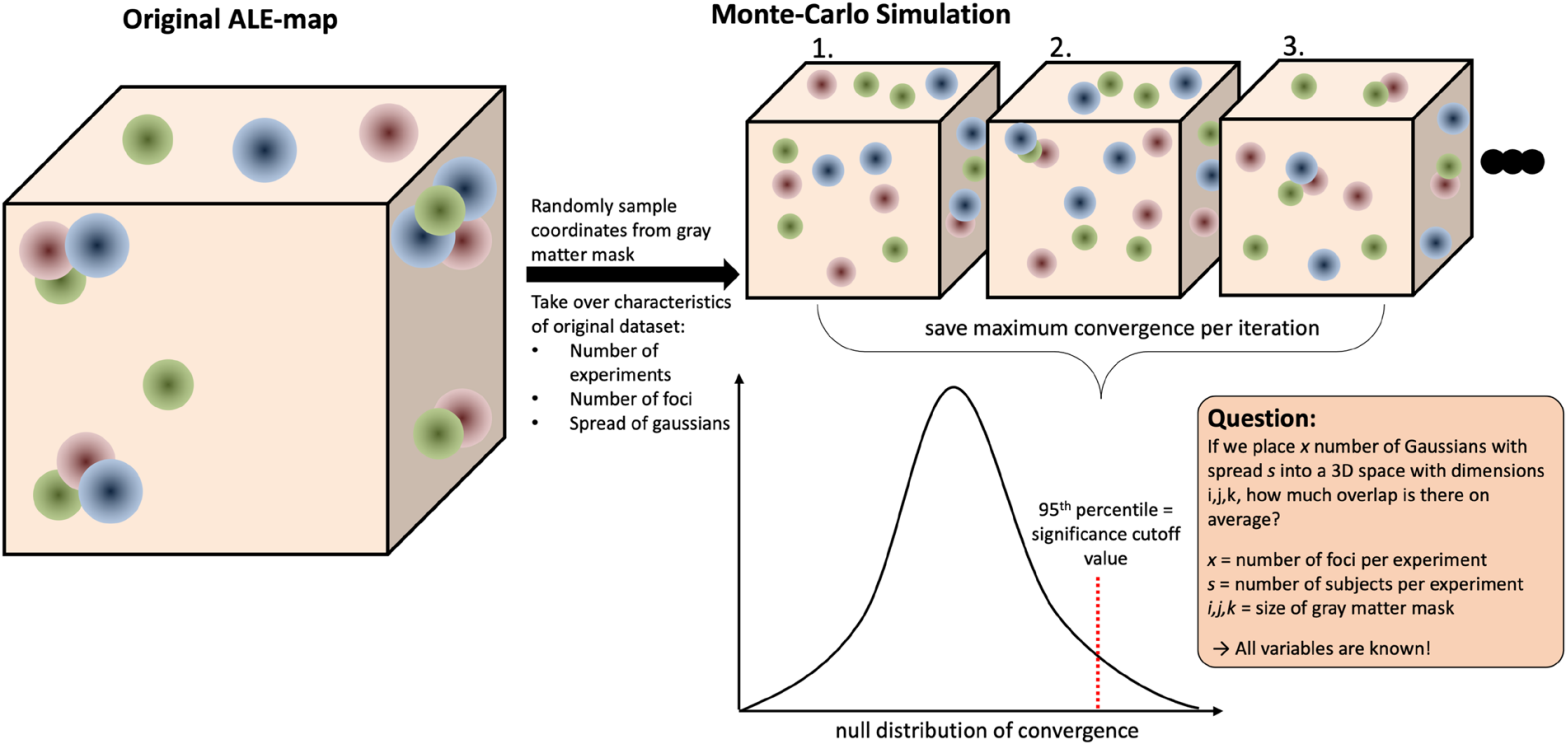
A simplified version of the Monte-Carlo simulation procedure employed in ALE. The brain is here represented by a cube and the foci of activation, already smoothed by 3-D Gaussians, are represented by colored circles. Each color represents a different experiment. First, the original ALE map is calculated based on the coordinates and experiment characteristics featured in the dataset. The dataset is then copied, and the coordinates randomly distributed in the brain. This is then repeated between 5000 and 10,000 times, and for each repetition the maximum convergence is saved. These saved maximum convergence values are then used as a null distribution against which the original (observed) convergence values are compared.

Activation likelihood estimation (ALE) is a widely used coordinate-based meta-analysis (CBMA) technique, which allows researchers to synthesize findings across multiple brain imaging studies (Laird, Fox, et al., 2005;Turkeltaub et al., 2002). ALE helps identify consistent patterns of brain activation by analyzing the spatial locations of activation foci reported in different studies. Importantly, ALE takes into account the spatial uncertainty inherent in neuroimaging results by modeling coordinates, representing peaks of activation, not as dimensionless points but as 3-D Gaussian probability distributions (Eickhoff et al., 2009). The smoothed results of all experiments are combined into an “ALE-map”, in which each voxel is assigned a value quantifying the between-experiment overlap observed. This between-experiment overlap is usually called “convergence” in the context of ALE meta-analyses. ALE then uses an analytical procedure, called non-linear histogram integration or convolution, to calculate a voxel-wise null distribution (Eickhoff et al., 2012). This procedure is extremely efficient and allows for the calculation of p-values and through these, significance testing on a voxel level. Unfortunately, due to the high number of statistical comparisons made on the whole-brain level, the chance of spuriously significant clusters (false-positives) is very high. Therefore, reporting uncorrected results is strongly discouraged. To control the rate of false positives, ALE traditionally employs voxel- or cluster-level family-wise error (vFWE or cFWE) correction (Eickhoff et al., 2012,^2016^). Recently, threshold-free cluster-enhancement (TFCE;Smith & Nichols, 2009) has been proposed as an alternative correction method (Frahm et al., 2022). All three correction algorithms are based on Monte-Carlo simulations, or permutation-based null distributions of spatial aggregation under the assumption of spatial independence of the coordinates (Fig. 1). In regard to implementation, this means making a copy of the original meta-analysis dataset but replacing the reported coordinates by coordinates randomly sampled from a gray-matter mask (>10% probability for gray matter;Evans et al., 1994). Next, a standard ALE is calculated for the random-association dataset, and the maximum amount of convergence is saved. The quantification of the amount of convergence depends on the correction algorithm: vFWE uses the highest ALE value, TFCE the highest TFCE-value, and cFWE uses the number of voxels in the largest continuous cluster after applying a cluster-forming threshold (at voxel-level). To get a good approximation of the distribution of maximum convergence found in random data (from here on: null distribution), this process needs to be repeated at least 1000 times, but, in general, it is recommended to use between 5000 to 10,000 permutations (Eickhoff et al., 2012). As a last step, the original ALE, z or TFCE-statistic map is thresholded against the 95^th^percentile of the null distribution, which corresponds to a p-value of 0.05. The value of this 95^th^percentile is the most relevant part of the null distribution and will hereafter be referred to as (significance) cutoff value. Through the cutoff value, the permutation procedure allows for null-hypothesis significance testing, while taking into account the number of statistical comparisons made. Even though the computations required for a single iteration of the permutation testing procedure are not particularly time intensive, computation time quickly accumulates when running thousands of iterations. This leads to an individual ALE analysis taking multiple hours, depending on the dataset and hardware used for running the computations.

The current project aimed to provide a machine-learning-based alternative to the permutation-based significance testing. To this end, we developed a method using a range of summary characteristics of the dataset (i.e., meta-data) as features to predict the cutoff value. This idea was inspired by the observation that the permutation-based testing procedure for any given dataset would always result in a specific null distribution with increasing repetitions. This means that for every ALE dataset there exists a deterministic cutoff value for each of the three thresholding techniques, vFWE, cFWE, and TFCE. These deterministic cutoff values differ between datasets, that is, there must be certain properties inherent to a given dataset that define the null distribution and, in turn, the cutoff value. The most vital part of any dataset collected for a coordinate-based meta-analysis is the coordinates reported by the different experiments, but as these coordinates get replaced by random coordinates for each permutation of the Monte-Carlo simulation, the original location of foci does not impact the null distribution obtained. To further corroborate this pivotal point, we ran Monte-Carlo simulations for 10 datasets that were identical regarding all characteristics but the location of their coordinates. As expected, the resulting cutoff values were the same or nearly identical for all 10 datasets (Supplementary Material). Furthermore, the random allocation of coordinates is the exact same for any dataset, meaning the sampling process also does not influence the cutoff value. This leaves the following dataset characteristics that shape the null distribution: the number of experiments, the number of subjects scanned in each experiment, and the number of foci reported by each experiment. Determining how these characteristics determine the null distribution might be solvable in a parametric way. However, the relationship between the parameters and the threshold is highly complex and therefore not solvable given our current mathematical tools, which is why we resorted to approximating the solution via machine-learning techniques.

**Fig. 2.**
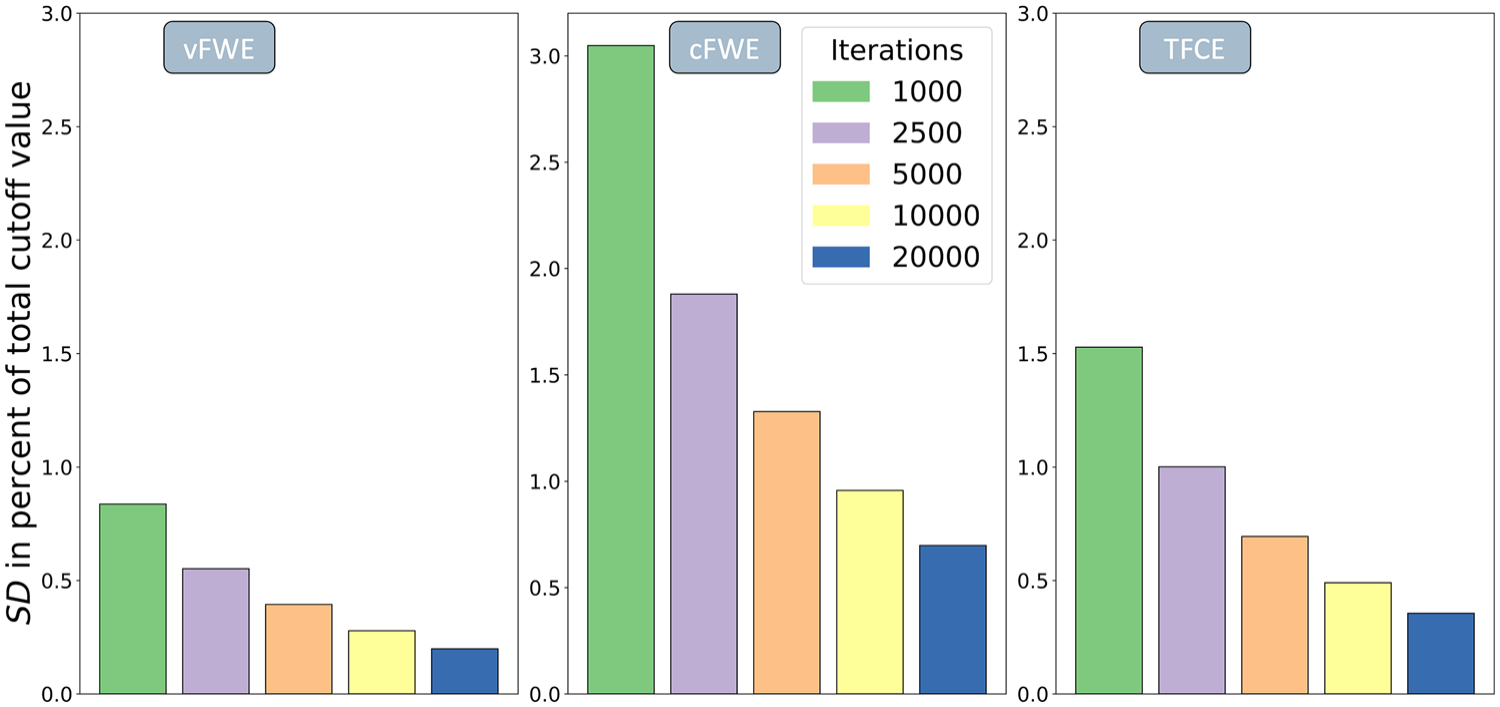
Variability in the results of the permutation-based thresholding procedure for different numbers of iterations. For assessment, we simulated a dataset with 35 experiments, calculated Monte-Carlo simulations with one million iterations, and took 10,000 slices of a certain size, representing the number of iterations. Each bar shows the standard deviation in percent of the total cutoff value for a certain number of iterations. The vFWE threshold shows the least amount of variance, while cFWE features the highest amount of variance.

Given the time-consuming nature of the permutation-based testing procedure, replacing it with an instantaneous prediction would be a major improvement for the ALE algorithm. This is especially true for more complex and advanced analysis set-ups, like jackknife or leave-one-out sensitivity analyses. These analyses are used to assess the stability of results, by running n (number of experiments) - 1 separate ALEs, excluding a different experiment in each run. Evidently, the time saved per ALE becomes much more impactful because it is multiplied by the number of analyses run. Another benefit of machine-learning predictions over the permutation-based testing procedure is replicability. For a given dataset, a trained regression model will always predict the same threshold, while the Monte-Carlo simulations approximate the “true cutoff” anew each time and therefore, depending on the number of iterations, show some rather substantial variance (Fig. 2).

In this study, we first simulated meta-analysis datasets, spanning a broad range of potential sizes and experiment characteristics. For all of these datasets, we ran extended Monte-Carlo simulations to approximate the true cutoff values for vFWE, cFWE, and TFCE as precisely as possible. We then trained multiple different machine-learning algorithms on this data using a 10-fold cross-validation scheme and lastly validated the best performing algorithm on 21 datasets from previously published ALE meta-analyses.

## 2 Methods

Our methodological set-up comprised four steps: (1) generating simulated training datasets, (2) running Monte-Carlo simulations to determine significance cutoff values for each simulated dataset, (3) training machine-learning models, evaluating their performance, and choosing the best performing model, and (4) validating model performance on “real-life” ALE datasets. As all analyses were performed on simulated or data freely provided by other authors, no additional approval by an ethics committee was required for this study.

### 2.1 Training data

As established in the introduction, the Monte-Carlo simulations are not dependent on the reported coordinates or convergence observed in the original (“real-life”) dataset. Therefore, technically it is possible to generate unlimited amounts of training data in the form of simulated meta-analysis datasets. The limiting factor in this case is the computation time required to run the Monte-Carlo simulations to get the cutoff values for a given dataset. To simulate a dataset, we chose a certain size (number of experiments) and then randomly sampled each experiment’s characteristics (number of subjects and number of foci) from a distribution of choice. Our aim was to expose the models to the broadest range of parameter combinations possible, to ensure that our models would be applicable to the wide range of datasets users might encounter in empirical research. In total, we simulated 68,100 datasets with 10 to 150 experiments, which encompasses the most frequently observed dataset sizes. The training data can be divided into four batches based on the distributions they are based on. The largest batch of datasets (50,000) were filled with experiments whose sample size and number of foci included were randomly sampled from normal distributions (sample size: mean = 20, standard deviation (SD) = 10; number of foci: mean = 15, SD = 10) similar to what is found in previously observed datasets according to the BrainMap database (Fox & Lancaster, 2002;Laird, Lancaster, et al., 2005). With the next batch we tried to model more heterogeneous datasets by sampling both parameters from uniform distributions (sample size: 5 to 50; number of foci: 1 to 30). This batch includes 9000 datasets. Through the third batch, we tried to model more extreme datasets by iterating over three distinct distributions (low, medium, high) for both sample size and number of foci, totaling nine combinations. For sample size, we used uniform distributions ranging from 4 to 10 subjects (low), from 10 to 25 subjects (medium), and from 25 to 50 subjects (high). For the number of foci, we used uniform distributions ranging from 1 to 5 foci (low), from 5 to 15 (medium), and from 10 to 30 foci (high). This batch included 6300 datasets. The last batch of training data modeled the most extreme of datasets, including experiments with up to 300 subjects or reporting up to 150 foci. This batch included 2800 datasets. After creating the datasets, we ran the ALE permutation testing procedure with 15,000 iterations per dataset to calculate the significance threshold for vFWE, cFWE, and TFCE, which served as ground-truth labels for training. Ideally, we would have used a much higher number of iterations (>100,000) per dataset to best approximate the ground truth (see alsoFig. 2). This, however, was not feasible due to the high computational demand of calculating so many permutations for almost 70,000 datasets. We decided that covering a broad range of potential dataset characteristics was more important than reducing the somewhat higher variability in the prediction response due to the limited number of permutations.

### 2.2 Features

When abstracting the question, the Monte-Carlo simulation tries to solve in ALE, it could be phrased like this: “If we randomly place*x*Gaussians with spread*s*into a 3D space with fixed dimensions, how much convergence will there be on average?” Following this question, the variables it contains, and the mathematical underpinnings of the ALE algorithm, it becomes clear which dataset characteristics influence the null distribution. The number of foci constitute*x,*the number of Gaussians. The spread*s*of these Gaussians is determined by the number of subjects. The number of experiments has a more indirect influence, based on the random-effects inference employed by ALE (Eickhoff et al., 2009). Based on this a priori assessment, we generated 26 features. The majority of the features are summary statistics about the number of participants and the number of foci per experiment, such as mean, median, standard deviation, minimum, maximum, skewness, and kurtosis. We additionally created more complex features. The first set of complex features comprised summary statistics over the ratio between the number of subjects and the number of foci. The second set of complex features divided the total number of foci into high impact, medium impact, low impact, and very low impact based on the number of subjects each experiment reported and summed them up per category.Table 1gives a complete overview over all features used.

**Table 1.** Features used for prediction.

Training data	
Name	Summary statistics
Number of experiments	-
Total number of foci / number of experiments	-
Number of subjects	Total
	Mean
	Median
	Standard deviation
	Maximum
	Minimum
	Skewness
	Kurtosis
Number of foci	Total
	Mean
	Median
	Standard deviation
	Maximum
	Minimum
	Skewness
	Kurtosis
Number of foci / number of subjects ratio	Mean
	Standard deviation
	Maximum
	Minimum
Number of foci by impact (number of subjects)	High impact (> 20)
	Medium impact (15 - 20)
	Low impact (10 - 15)
	Very low impact (< 10)

For some features, aggregation/ summary statistics were necessary to keep the number of features stable and independent from the dataset size. In total, we used 26 features.

### 2.3 Evaluation and model selection

We trained and evaluated 6 different regression models (linear regression, ridge regression, k-nearest neighbor regression (KNN), RandomForest, AdaBoost, and XGBoost) (Pedregosa at al., 2011) using their default hyperparameter values as implemented in Scikit-learn and the XGBoost python package (Chen & Guestrin, 2016). For a more detailed description, please refer to theSupplementary Material. Model evaluation was based on a 10-fold cross-validation scheme using the complete set of simulated datasets. This means we always trained the models on 61,290 datasets and predicted the cutoff value for the remaining 6810 held-out datasets. We used mean absolute percentage error (MAPE) and the coefficient of determination (R²) averaged over all folds as performance metrics. The best performing model, based on its R² score, per thresholding technique was then validated on real-life datasets.

### 2.4 Validation on published datasets

Even though the simulated datasets were created in a way that aimed to cover the broad range of possible dataset characteristics as much as possible, it is important to confirm that the models perform well on real-life ALE datasets. To this end, we trained three selected models (one per threshold type) on all 68,100 simulated datasets and predicted thresholds in 11 previously published (or currently reviewed) ALE datasets, across 21 different contrasts (Table 2). The real-life ALE datasets spanned a large range of different sizes, subject populations, and cognitive domains and should therefore be largely representative of the majority of future ALEs. We then compared the predicted thresholds to thresholds calculated by permutation testing with 50,000 iterations. We increased the number of iterations to this level to ensure that we would approximate the underlying distribution as closely as possible.

**Table 2.** Datasets and contrasts used for empirical model validation.

Datasets & Contrasts				
Author (Year)	Domain	Modality	Contrast	Number of experiments
Langner and Eickhoff (2013)	Sustained Attention	TA	All	67
Kogler et al. (2015)	Stress	TA	All	125
			Physical	82
			Social	43
Müller et al. (2017)	Depression	TA	All	99
			Cognition	34
			Emotion	65
			Activation	50
			Deactivation	49
Kogler et al. (2020)	Empathy	TA	Affective empathy	19
			Cognitive empathy	38
			Empathy for pain	24
			Empathy for emotions	33
			Pain	72
Henn et al. (2022)	Chronic Pain	VBM	All	103
Kamalian et al (2022)	Dementia	VBM/VBP	All	31
Rahimi-Jafari et al. (2022)	Narcolepsy	TA/VBM	All	15
Saberi et al. (2022)	Late-life depression	VBM/VBP	All	26
Naghibi et al. (2023)	Time perception	TA	All	95
Cieslik et al. (2023)	Task control	TA	All	143
Reimann et al. (under review)	Insomnia	VBM/VBP	All	26

For some datasets we used multiple contrasts, which allowed us to get a larger range of dataset sizes without having to acquire additional full datasets. All contrasts used are or will be part of an ALE meta-analysis publication. In the modality column, TA stands for task-activation, VBM for voxel-based morphometry, and VBP for voxel-based physiology.

## 3 Results

### 3.1 Prediction performance and model selection in simulated data

**Fig. 3.**
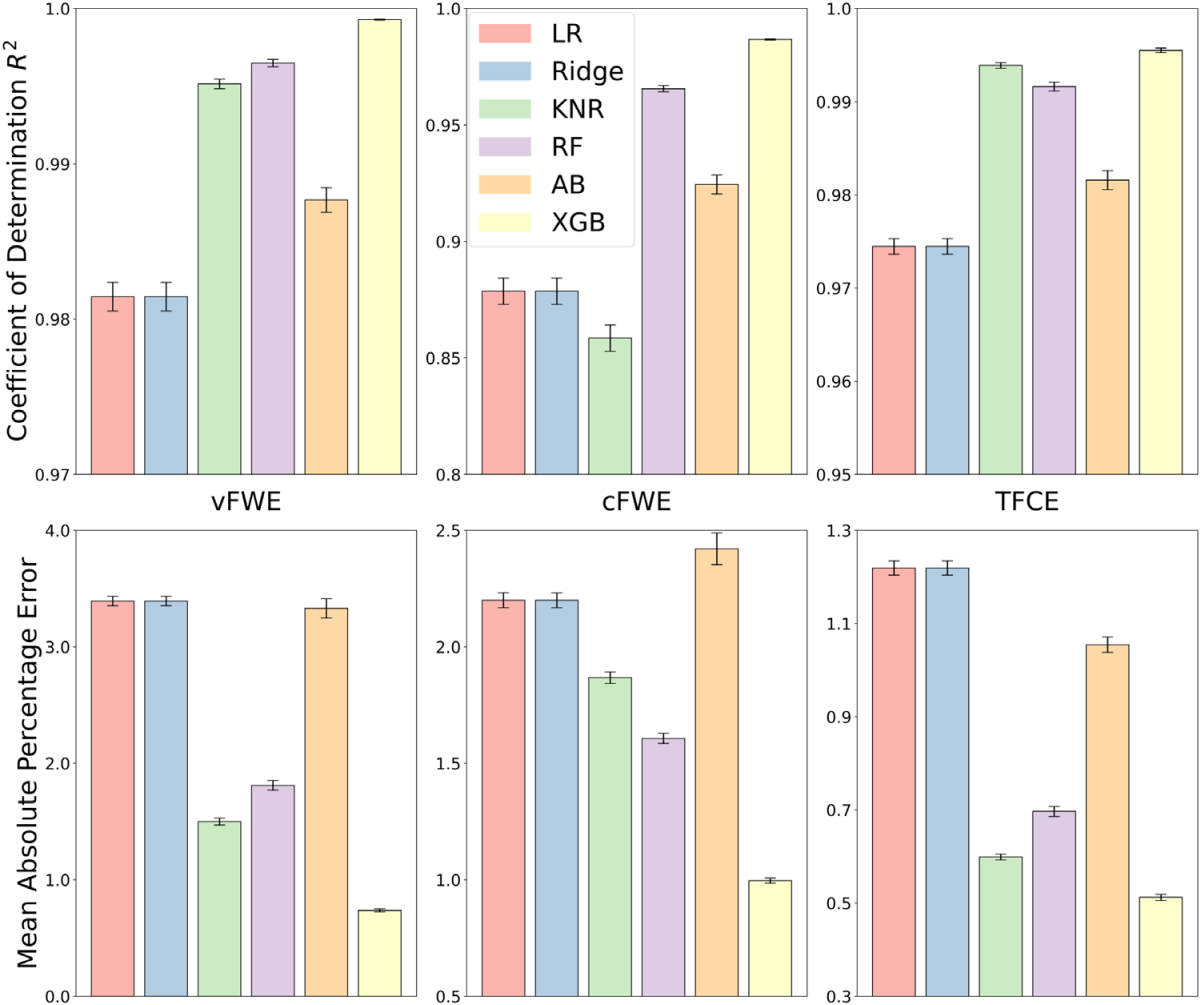
Prediction performance (as indicated by R² and MAPE) of different regression algorithms when using a 10-fold cross-validation scheme on the full training data. Left: prediction of the vFWE threshold. XGBoost performed best, closely followed by RandomForest and K-nearest neighbor regression. Middle: prediction of the cFWE threshold. XGBoost performed best, closely followed by RandomForest. Right: prediction of the TFCE threshold. XGBoost performed best, closely followed by K-nearest neighbor and RandomForest.

Regarding the prediction of vFWE cutoff values in the simulated data, all models performed at an extremely high level (Fig. 3). The worst performing models were linear regression, ridge regression, and AdaBoost but still with high average R² values between 0.983 and 0.985. Both KNN and RandomForest performed slightly better with average R² values of 0.994 and 0.996, respectively. The best-performing model was XGBoost with an R² of 0.999, which basically constitutes a perfect prediction. The performance of models when predicting cFWE cutoff values was slightly worse than what was observed for vFWE cutoffs, but still at a very good level. The worst performing algorithm was KNN, which achieved an R² of 0.850. Linear regression, ridge regression, and AdaBoost performed better with R² scores between 0.923 and 0.933. As with vFWE cutoff prediction, the best performing models were RandomForest (R² = 0.967) and XGBoost (R² = 0.986). The prediction of TFCE thresholds was similar in accuracy and ranking of models to the prediction of vFWE thresholds. The worst performing models were linear and ridge regression, each yielding an R² of 0.974, only slightly surpassed by AdaBoost at 0.981. RandomForest, KNN, and XGBoost all produced R² scores above 0.99. For all three thresholding methods, XGBoost was able to capture the relationship between dataset characteristics and the cutoff values the best, and it did so while being one of the computationally faster algorithms and without any hyperparameter tuning. We, therefore, decided to use XGBoost for all three threshold types and proceed with our validation.

We also analyzed the prediction performance based on the mean absolute percentage error (MAPE), which represents the size of the prediction errors relative to the simulation-derived mean values in terms of percentages. Of note, although all models performed with very high R2 (>0.98) when predicting the vFWE threshold, the MAPE was comparatively large (>3%). Nonetheless, XGBoost performed best on this metric as well, featuring MAPE values of about 0.5 for vFWE, 0.9 for cFWE, and 0.5 for TFCE.

### 3.2 Validation in real-life data

**Fig. 4.**
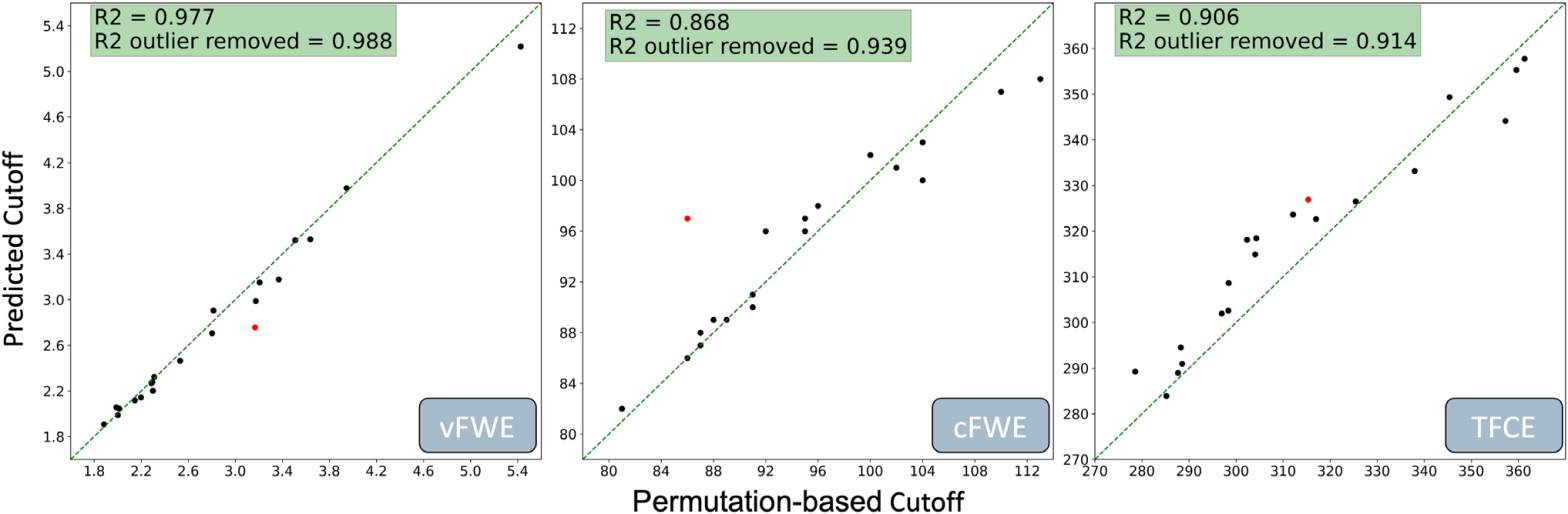
Prediction performance of the XGBoost regression for vFWE (left), cFWE (middle) and TFCE thresholds for previously unseen naturalistic datasets. The regression line was added for illustrating a perfect linear correspondence between the two. The red data point indicates a dataset that falls outside the parameter space covered by the simulated training data, featuring the largest prediction error for both vFWE and cFWE models.

The algorithm was able to predict all three significance thresholds in unseen naturalistic datasets with very high accuracy for vFWE (R² = 0.985), cFWE (R² = 0.882), and TFCE (R² = 0.95) (Fig. 4). It can be observed that there is an order in performance following the abstraction level of the cutoff value. The vFWE cutoff value is a voxel-based ALE value and is therefore most immediately connected to the dataset parameters. The TFCE cutoff, though still at the voxel level, is derived from a z-statistic—one step abstracted from ALE values. Accordingly, its predictive performance was slightly lower. The lowest direct correlation was observed for the cFWE threshold, which determines a minimum cluster size after a voxel-level inclusion thresholding based on a z-statistic. This more indirect relationship between dataset characteristics and the cFWE threshold was reflected in its relatively lower prediction accuracy.

Notably, there is one dataset (Kamalian et al., 2022) which showed the by far largest prediction error for both vFWE and cFWE (shown inFig. 4as a red dot). In this dataset, there are two experiments which feature 634 and 175 foci, respectively, due to the fact that the authors had to aggregate numerous studies that used the same sample of participants (large-scale public dataset) and therefore could not be counted as independent experiments in the ALE analysis. These high numbers of foci are very unusual and exceed the maximum number of foci for experiments in the simulated training datasets, which was 150. The larger prediction error for this dataset showed that even though the prediction algorithms performed very well on unseen datasets that fall into the expected parameter space, they are not able to extrapolate, which is not unexpected. When removing this dataset from the validation, the R² score for the vFWE prediction increased to 0.996, while it increased to 0.951 for the cFWE prediction. It should be noted that the TFCE threshold prediction for this dataset is very good. Therefore, removing the dataset does not lead to a notable increase in overall TFCE prediction accuracy.

We additionally examined the size and center positions of the significant clusters resulting from either the predicted threshold or the threshold based on extended (100,000 permutations) Monte-Carlo simulations (Table 3 inSupplementary Material). In total, we analyzed 20 different contrasts with three thresholding techniques, resulting in 60 analyses overall. For vFWE, 12 out of 20 analyses using the predicted threshold produced identical results compared to those observed with the simulation-derived threshold. Seven analyses revealed small changes in size (<30 voxels in total or <10% total voxel count) and in the center positions of some significant clusters (<4 mm change in centers). When analyzing the task control dataset fromCieslik et al. (2023), cluster 11 was detected only with the application of the Monte-Carlo threshold. Upon further investigation, we found that the slightly lower predicted threshold caused cluster 11 to merge with cluster 3, forming a single larger cluster.

Predicting cFWE thresholds resulted in identical findings in 18 out of 20 analyses. In the remaining two cases, individual clusters were one and two voxels short of the cluster extent threshold used by cFWE, respectively. These discrepancies represent extreme edge cases, as even a standard Monte-Carlo simulation running 5000 to 10,000 permutations would have an equal probability of the clusters being accepted or rejected. TFCE thresholding demonstrated a performance similar to vFWE, with 11 analyses producing identical results, 8 showing minor changes, and 1 analysis revealing the breakup of one large cluster into three subclusters.

### 3.3 Feature Importance

**Fig. 5.**
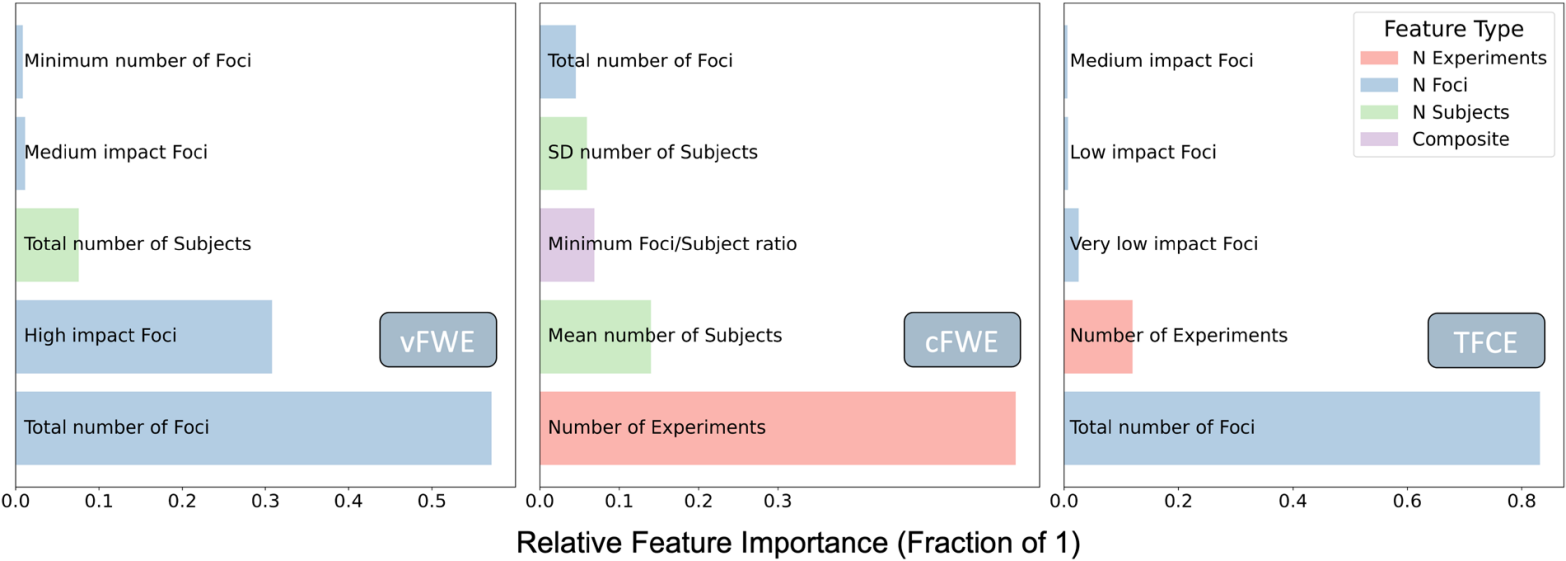
Feature importance of the three XGBoost models trained on the full training dataset. Red features are based on the number of experiments, blue features on the number of foci, green features on the number of subjects and purple features are composite features made up out of a combination of the three feature types listed above. It can be observed that vFWE and TFCE are both largely influenced by the number of foci, while cFWE is influenced more by the number of experiments and the number of subjects.

We engineered features based on prior knowledge of the ALE algorithm and intuition. Looking at the feature weights of our final models allowed us to learn more about the association between dataset characteristics and the outcome of the Monte-Carlo simulation (Fig. 5). Inspecting the features that drive the predictions for each of the thresholding techniques showed that the TFCE threshold seems to be almost exclusively influenced by the total number of foci in the dataset. This is similar to the voxel-level cutoff, which is also strongly driven by the total number of foci but is additionally influenced by the number of high-impact foci (experiments >20 subjects). For the prediction of the cFWE cutoff-value, the model is using a much broader array of features. The most important feature is the number of experiments in the dataset followed by the average number of subjects. The next four features, namely the minimal ratio of foci to subject, the total number of foci, the standard deviation of the number of subjects, and the total number of very low impact foci (experiments <10 subjects), all still contribute majorly to the prediction (>5% contribution). It should be noted that none of the features had a feature importance of 0, which means that even though some of the features are highly correlated, they all seem to capture some unique variance of the cutoff value. This is why we did not further reduce the feature space to achieve a simpler model.

## 4 Discussion

The current study aimed to provide an alternative to the time-consuming Monte-Carlo simulations ALE employs to estimate significance thresholds corrected for multiple comparisons with machine-learning-based predictions. To achieve this, we simulated close to 70,000 ALE datasets, spanning a large range of potential different size and experiment characteristics. We performed extensive Monte-Carlo simulations (15,000 iterations per analysis) on these datasets to determine the “true” significance thresholds. Using dataset characteristics as features, like the average number of subjects or the total number of foci, we trained machine-learning algorithms to predict the “true” significance thresholds for both vFWE and cFWE. We selected the most appropriate algorithm by running a 10-fold cross-validation scheme. We then continued to validate the best-performing algorithm on real-life ALE datasets. These datasets were taken from previously published or submitted ALE meta-analyses (Table 2), which therefore constitute a highly realistic empirical validation set. As a last step, we compared the computation time required for the Monte-Carlo simulations in the validation datasets to that required for the predictions. In general, the prediction of vFWE, cFWE, and TFCE cutoff values worked extremely well. Using XGBoost (Chen & Guestrin, 2016) with its standard parameters, we were able to achieve R²-scores of 0.996 for vFWE thresholds, 0.939 for cFWE thresholds, and 0.953 for TFCE thresholds in previously unseen real-life datasets. Replacing the permutation testing by instantaneous predictions can save between 1 to 5 hours depending on the dataset size for a singular ALE analysis.

### 4.1 Approximation of true cutoff values

The Monte-Carlo simulation procedure currently used in ALE only approximates the null distribution of spatial convergence, which leads to variance of the determined (and to-be-predicted) cutoff value (Fig. 2). Interestingly, the three thresholding techniques differ in their cutoff value variance when keeping the permutations constant. This difference seems to be based on the thresholding technique’s level of abstraction or complexity. vFWE, which is directly calculated from ALE scores, features the lowest variance, while cFWE which is based on an initial ALE score–based thresholding and a subsequent cluster size evaluation, shows the highest variance. The different levels of abstraction can also later be observed in the prediction performances for each technique, with thresholds obtained from more complex techniques being harder to predict. An additional way in which the variance of the determined cutoff value impacts the performance of the models is that the algorithm will be presented with an approximated label during training. This can lead to the algorithm learning slightly wrong associations between features (i.e., dataset characteristics) and the target variable (i.e., the significance threshold). One possible solution would have been to increase the number of iterations calculated. Due to the high computational cost connected with the Monte-Carlo simulations, we had to decide between using fewer datasets with more permutation iterations (smaller parameter space coverage, higher cutoff precision) or more datasets with fewer permutation iterations (larger parameter space coverage, lower cutoff precision). Considering this trade-off, we decided to only slightly increase the number of iterations from the values recommended in the literature, which allowed us to focus on covering as much of the possible dataset space as possible. Our results confirm that this approach worked as the prediction error caused by datasets with characteristics outside the parameter space used in training was much larger than the imprecision caused by the approximated cutoff value. In general, it should be noted that both the variance inherent in the permutation procedure at 5000 to 10,000 iterations and the prediction error observed in the validation datasets are negligible in the grand scheme of things and should not influence the results of a given ALE analysis to a notable degree. This is especially true because the variance is not systematic but random and it is therefore equally likely to get a slightly lower or higher threshold.

### 4.2 Out-of-distribution prediction

Our models were able to predict unseen data with high accuracy. The only exception was a real-life ALE dataset which included experiments that reported many more foci than any simulated experiment we included in our training data. The lackluster accuracy for this dataset is not surprising as such out-of-distribution (OOD) predictions are a common problem in the realm of machine learning and statistical modeling (Amodei et al., 2016). There are two main ways of dealing with such sample anomalies. The first is building a model which generalizes well even to OOD samples using complex training mechanisms (e.g.,Yi et al., 2021). The second is detecting samples which go beyond the distributions encountered in the training data (e.g.,Yang et al., 2021) and then either modifying or rejecting the prediction and allowing for human intervention. This second approach is preferred in situations that require high prediction accuracy, which is why we decided to follow it. In comparison to many other domains in which such out-of-distribution detection is applied, we have one major advantage: we know the exact range of feature distributions present in the training data. We were, therefore, able to define boundary conditions for which we could “guarantee” the promised prediction accuracies. In particular, to be considered eligible for our prediction-based thresholding, meta-analysis datasets need to comprise between 10 and 150 experiments, with no experiment having more than 300 subjects and no experiment reporting more than 150 foci. In our implementation, a warning is shown to the user when recognizing datasets with characteristics that violate these boundary conditions and the standard permutation-based testing is run instead. The predicted threshold is then used to indicate early stopping, in case the predicted and the approximated cutoff values converge after 1000 (2000, 3000, etc.) iterations. With the number of neuroimaging publications growing each year and the steadily increasing sample sizes, the range of training data might need to be extended at some point to ensure compatibility with future ALE datasets.

### 4.3 Prediction-based thresholding beyond ALE

Other CBMA approaches, most notably seed-based d mapping (SDM-PSI;Albajes-Eizagirre et al., 2019), also use Monte-Carlo simulation or permutation testing set-ups to control the family-wise error rate. Even though the algorithmic procedures may be slightly different, there should be enough similarities to warrant a thorough look into the possibility of using a similar threshold prediction approach as described here for ALE. Additionally, there are a multitude of neuroimaging domains, besides meta-analyses, in which Monte-Carlo simulations are employed, for some of which a replacement with a prediction algorithm could be a potential improvement. A brief literature search did not uncover any previous attempts at this, which makes it even more important for future research to investigate possibilities in this direction.

### 4.4 Future of ALE using cutoff predictions

Reducing the computation time for individual ALE analyses from hours to minutes is a significant advance but may appear less important when considering the lengthy process of manually curating meta-analysis datasets, which can take up to months. This reduction in compute time is, nevertheless, crucial for the future of ALE for multiple reasons. First, ALE meta-analyses often involve running numerous contrasts, each with different inclusion criteria, sampling from the whole dataset. For example,Müller et al. (2017)ran 16 different ALEs in their study of altered brain activity in unipolar depression. Second, the recent trend of supplementing ALE with jackknife analysis, as seen in studies likeSong et al. (2021)andTablante et al. (2023), requires recomputing the ALE multiple times for leave-one-out cross-validation. This process, essential for assessing the reliability of ALE results, demands running at least as many ALE analyses as there are experiments in the dataset. These factors highlight the ongoing need for faster ALE computation, a need that will only grow as more complex applications of ALE emerge.

## 5 Conclusion

ALE employs vFWE, cFWE, or TFCE corrections to allow for testing statistical significance corrected for multiple comparisons. These corrections are based on Monte-Carlo simulations through which a null distribution of spatial convergence across experiments is approximated. The 95^th^percentile of this null distribution is then used as a significance threshold for the ALE maps resulting from the original dataset. The only major downside of this methodology is the high computation time, with runtimes of up to several hours. In this study, we demonstrated that ALE significance thresholds can be predicted with extremely high accuracy using XGBoost regression models based on features derived from a few characteristics and summary statistics of the meta-analysis dataset. As these predictions are nearly instant, our approach is able to save hours of computation time per ALE analysis without losing a relevant amount of thresholding accuracy. We, therefore, recommend replacing the Monte-Carlo simulations with predictions based on our models for future ALE analyses.

## Supplementary Material

Supplementary Material

## Data Availability

The code for this project is available athttps://github.com/LenFrahm/ALE_cutoff_prediction. The underlying data are only available on request due to storage limitations and privacy issues.
