# Correction to: Predictive modeling of significance thresholding in activation likelihood estimation meta-analysis

**DOI:** 10.1162/IMAG.x.934

**Published:** 2025-10-03

**Authors:** Lennart Frahm, Kaustubh R. Patil, Theodore D. Satterthwaite, Peter T. Fox, Simon B. Eickhoff, Robert Langner

**Affiliations:** Department of Psychiatry, Psychotherapy and Psychosomatics, School of Medicine, RWTH Aachen University, Aachen, Germany; Institute of Neuroscience and Medicine (INM7: Brain and Behavior), Research Centre Jülich, Jülich, Germany; Institute of Systems Neuroscience, Medical Faculty, Heinrich Heine University, Düsseldorf, Germany; Department of Psychiatry, Perelman School of Medicine, University of Pennsylvania, Philadelphia, PA, United States; Penn Lifespan Informatics and Neuroimaging Center, Perelman School of Medicine, University of Pennsylvania, Philadelphia, PA, United States; Research Imaging Institute, University of Texas Health Science Center, San Antonio, TX, United States; Departments of Radiology, Neurology, Psychiatry and Behavioral Sciences, and Physiology, University of Texas Health Science Center, San Antonio, TX, United States

In the original publication there was an error in the Author Contributions. “L.F. and R.L. came up with the first drafts of the paper” should read “L.F. wrote the first draft of the manuscript and R.L. provided feedback.”

