# Supplementary material for: Predictive modeling of significance thresholding in activation likelihood estimation meta-analysis
Source: Imaging Neurosci (Camb). 2025 Jan 10;3:imag_a_00423. doi: 10.1162/imag_a_00423 (PMC12319800; doi:10.1162/imag_a_00423)
Supplement: Supplementary Material [file imag_a_00423-supp.pdf]

Supplementary Material

Machine learning model parameters:

All models are sourced from https://scikit-learn.org/stable/ except XGBoost https://xgboost.readthedocs.io/en/stable/

1. Linear Regression (linear_model.LinearRegression)
    - fit_intercept: True
    - normalize: False
2. Ridge Regression (linear_model.Ridge)
    - alpha: 1.0
    - fit_intercept: True
    - normalize: False
    - max_iter: None
    - tol: 1e-3
    - solver: 'auto'
3. K-Nearest Neighbors Regression (neighbors.KNeighborsRegressor)
    - n_neighbors: 5
    - weights: 'uniform'
    - algorithm: 'auto'
    - leaf_size: 30
    - p: 2
    - metric: 'minkowski'
    - metric_params: None
4. Random Forest (ensemble.RandomForestRegressor)
    - n_estimators: 100
    - criterion: 'squared_error'
    - max_depth: None
    - min_samples_split: 2
    - min_samples_leaf: 1
    - min_weight_fraction_leaf: 0.0
    - max_features: 'auto'
    - max_leaf_nodes: None
    - min_impurity_decrease: 0.0
    - bootstrap: True
    - oob_score: False
    - random_state: None
    - ccp_alpha: 0.0
5. AdaBoost (ensemble.AdaBoostRegressor)
    - base_estimator: None
    - n_estimators: 50
    - learning_rate: 1.0

loss: 'linear'
random_state: None

6. XGBRegressor
   objective: 'reg:squarederror'
   base_score: 0.5
   booster: 'gbtree'
   colsample_bylevel: 1
   colsample_bynode: 1
   colsample_bytree: 1
   gamma: 0
   gpu_id: -1
   importance_type: 'gain'
   interaction_constraints: ''
   learning_rate (eta): 0.3
   max_delta_step: 0
   max_depth: 6
   min_child_weight: 1
   missing: None
   n_estimators: 100
   n_jobs: 1
   num_parallel_tree: 1
   predictor: 'auto'
   random_state: 0
   reg_alpha (alpha): 0
   reg_lambda (lambda): 1
   scale_pos_weight: 1
   subsample: 1
   tree_method: 'auto'
   validate_parameters: 1

Independence of cutoff-value from position of foci – Additional Analysis

10 Datasets with same characteristics:

- Number of Experiments = 30
- Number of Subjects for all Experiments = 15
- Number of Foci for all Experiments = 10
- Random foci, sampled from grey matter mask

100’000 permutations Monte-Carlo simulation
Kruskal-Wallis Test over all 10 cutoff-value distributions

Thresholding Technique	Kruskal-Wallis H-statistic	p-value
vFWE	8.37	0.5

cFWE	5.56	0.78
TFCE	12.5	0.19

Distributions vFWE

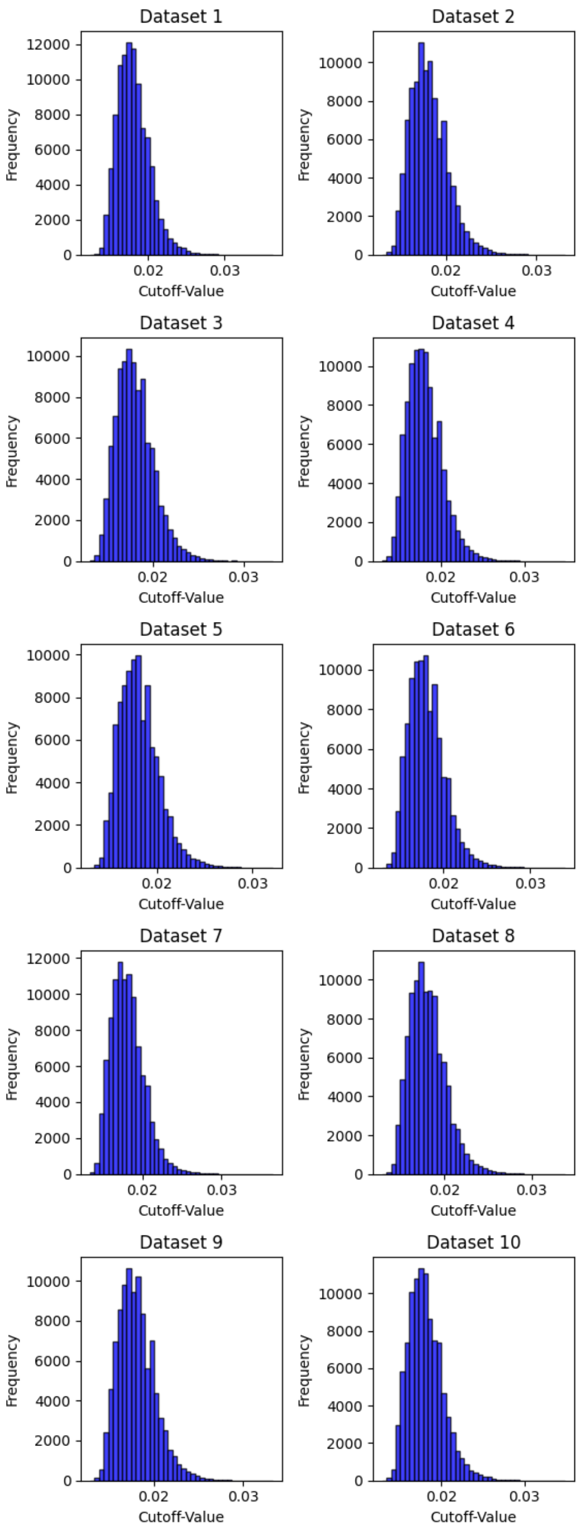
Dataset 1
Dataset 2
Dataset 3
Dataset 4
Dataset 5
Dataset 6
Dataset 7
Dataset 8
Dataset 9
Dataset 10
Frequency
Cutoff-Value
0
2000
4000
6000
8000
10000
12000
0.02
0.03

Distributions cFWE

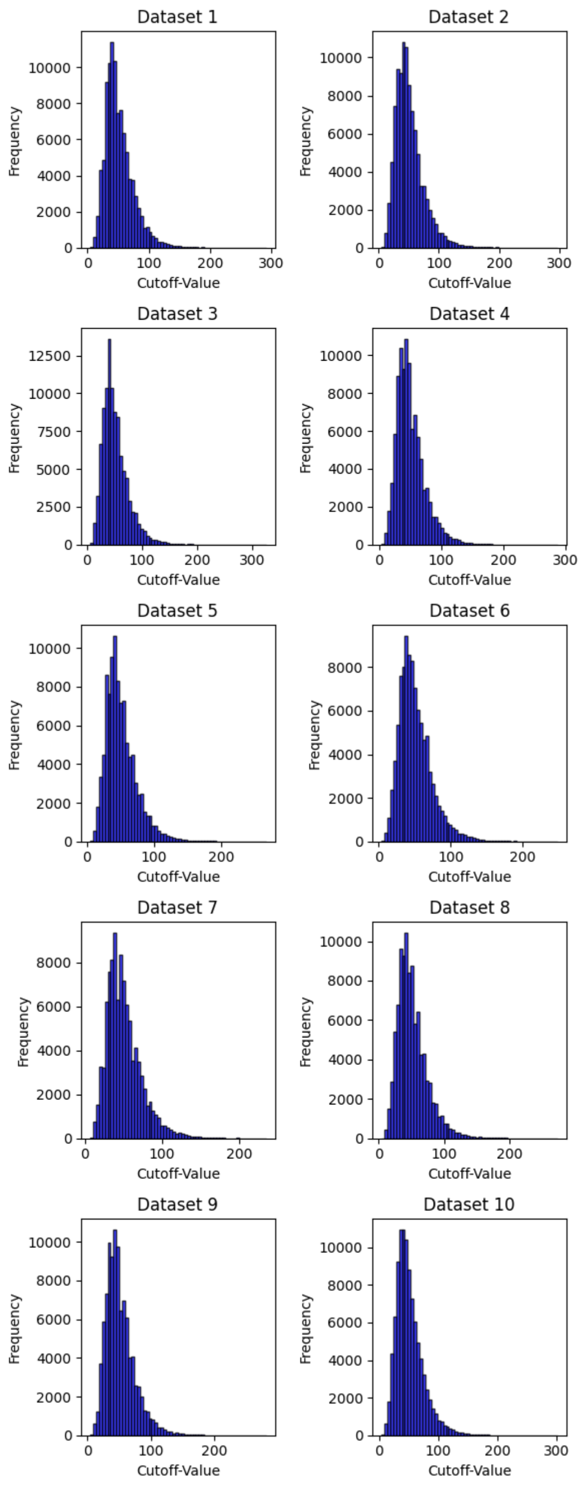

Distributions TFCE

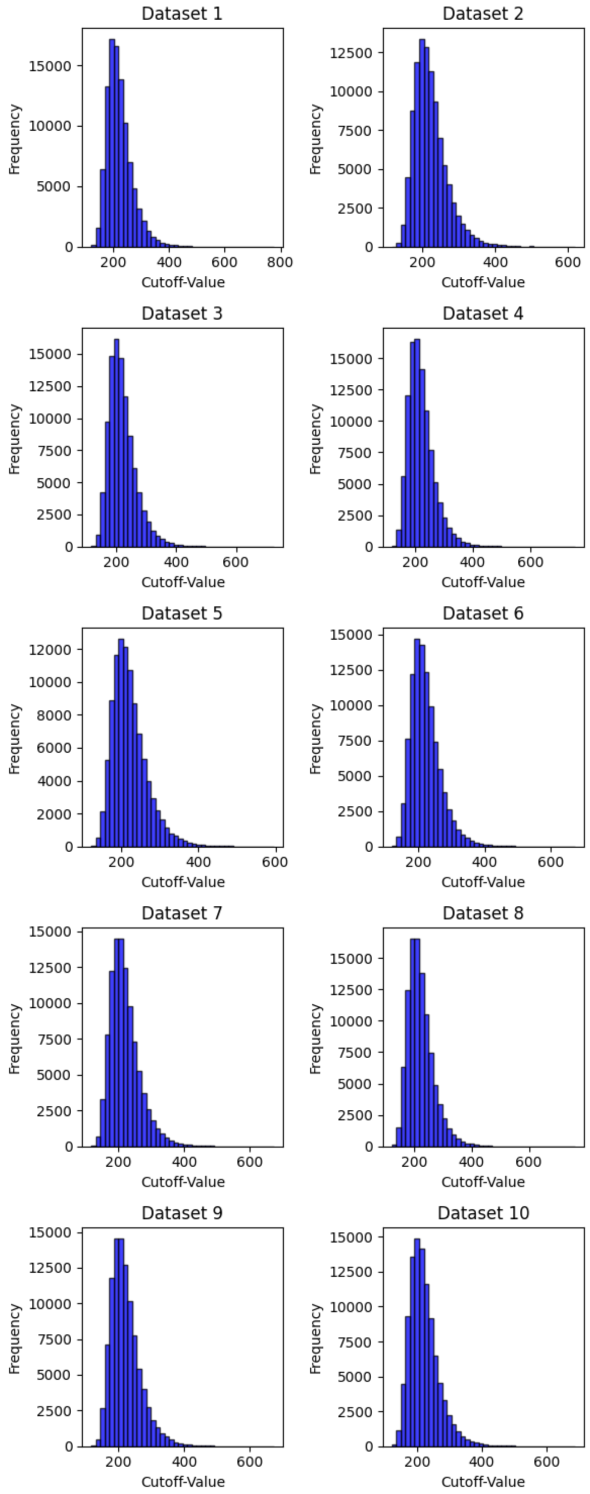

Validation of real-life Datasets

Table 3. Comparing the significant clusters extracted from the real-life datasets with the three multiple-comparison correction techniques based on either Monte-Carlo simulation-derived thresholds or predicted thresholds. Dark green color indicates that the clusters found were identical, lighter green indicates a small change in size or center location, yellow indicates a larger change in size or center, and red indicates a cluster does not achieve significance for either the Monte-Carlo or the predicted threshold.

Dementia		
	**vFWE MonteCarlo**	**vFWE Prediction**
Cluster 1	42 Voxel [Center: 26/-6/-14]	73 Voxel [Center 26/-8/-14]
Cluster 2	17 Voxel [Center: 0/36/-10]	39 Voxel [Center: 0/36/-10]
	**cFWE MonteCarlo**	**cFWE Prediction**
Cluster 1	633 Voxel [Center: -6/12/-6]	633 Voxel [Center: -6/12/-6]
Cluster 2	404 Voxel [Center: -2/38/-20]	404 Voxel [Center: -2/38/-20]
Cluster 3	248 Voxel [Center: -38/18/2]	248 Voxel [Center: -38/18/2]
Cluster 4	247 Voxel [Center: 28/-8/-16]	247 Voxel [Center: 28/-8/-16]
Cluster 5	163 Voxel [Center: 0/36/-10]	163 Voxel [Center: 0/36/-10]
Cluster 6	114 Voxel [Center: -4/40/-24]	114 Voxel [Center: -4/40/-24]
	**TFCE MonteCarlo**	**TFCE Prediction**
Cluster 1	9763 Voxel [Center: -2/26/6]	7911 Voxel [Center: -4/28/6]
Cluster 2	262 Voxel [Center: 28/-8/-16]	232 Voxel [Center: 28/-8/-16]
Cluster 3	36 Voxel [Center: -40/-23/-17]	373 Voxel [Center: 14/12/6]
Cluster 4		86 Voxel [Center: -56/8/-22]
Cluster 5		64 Voxel [Center: 28/36/-16]

Task Control		
	**vFWE MonteCarlo**	**vFWE Prediction**
Cluster 1	1529 voxel [Center: 44/18/4]	1577 voxel [Center: 44/18/4]
Cluster 2	812 voxel [Center: 4/18/44]	837 voxel [Center: 4/18/44]
Cluster 3	731 voxel [Center: -42/-46/44]	821 voxel [Center: -40/-48/44]
Cluster 4	525 voxel [Center: -34/18/-2]	536 voxel [Center: -34/18/-2]
Cluster 5	365 voxel [Center: 38/-48/46]	386 voxel [Center: 38/-46/46]
Cluster 6	342 voxel [Center: -44/8/32]	350 voxel [Center: -44/8/32]
Cluster 7	261 voxel [Center: 60/-44/22]	281 voxel [Center: 60/-44/22]
Cluster 8	230 voxel [Center: 12/4/6]	245 voxel [Center: 12/2/6]
Cluster 9	84 voxel [Center: 42/38/22]	94 voxel [Center: 40/38/22]
Cluster 10	83 voxel [Center: -26/-4/56]	90 voxel [Center: -26/-4/56]
Cluster 11	62 voxel [Center: -26/-66/36]	
Cluster 12	61 voxel [Center: -42/-68/-6]	66 voxel [Center: -42/-67/-7]

Cluster 13	48 voxel [Center: 36/2/54]	52 voxel [Center: 36/2/54]
	**cFWE MonteCarlo**	**cFWE Prediction**
Cluster 1	4122 voxel [Center: 42/18/14]	4122 voxel [Center: 42/18/14]
Cluster 2	2577 voxel [Center: 50/-44/36]	2577 voxel [Center: 50/-44/36]
Cluster 3	2301 voxel [Center: -42/-50/42]	2301 voxel [Center: -42/-50/42]
Cluster 4	1924 voxel [Center: 4/18/46]	1924 voxel [Center: 4/18/46]
Cluster 5	1051 voxel [Center: -44/12/30]	1051 voxel [Center: -44/12/30]
Cluster 6	1017 voxel [Center: -34/18/-2]	1017 voxel [Center: -34/18/-2]
Cluster 7	754 voxel [Center: 12/-2/6]	754 voxel [Center: 12/-2/6]
Cluster 8	538 voxel [Center: -44/-66/-10]	538 voxel [Center: -44/-66/-10]
Cluster 9	334 voxel [Center: -28/-4/56]	334 voxel [Center: -28/-4/56]
Cluster 10	288 voxel [Center: -12/0/6]	288 voxel [Center: -12/0/6]
Cluster 11	133 voxel [Center: 10/-68/48]	133 voxel [Center: 10/-68/48]
	**TFCE MonteCarlo**	**TFCE Prediction**
Cluster 1	8638 voxel [Center: 40/16/12]	8638 voxel [Center: 40/16/12]
Cluster 2	3899 voxel [Center: 50/-44/36]	3899 voxel [Center: 50/-44/36]
Cluster 3	2880 voxel [Center: 4/18/48]	2880 voxel [Center: 4/18/48]
Cluster 4	2861 voxel [Center: -44/-50/42]	2861 voxel [Center: -44/-50/42]
Cluster 5	2212 voxel [Center: -30/14/2]	2212 voxel [Center: -30/14/2]
Cluster 6	1697 voxel [Center: -44/14/30]	1697 voxel [Center: -44/14/30]
Cluster 7	540 voxel [Center: -30/-4/56]	540 voxel [Center: -30/-4/56]
Cluster 8	453 voxel [Center: -44/-66/-10]	453 voxel [Center: -44/-66/-10]
Cluster 9	71 voxel [Center: 48/-70/-2]	71 voxel [Center: 48/-70/-2]
Cluster 10	60 voxel [Center: 40/-86/4]	60 voxel [Center: 40/-86/4]
Cluster 11	41 voxel [Center: 20/-30/-2]	41 voxel [Center: 20/-30/-2]

**Insomnia**		
	**vFWE MonteCarlo**	**vFWE Prediction**
No Clusters		
	**cFWE MonteCarlo**	**cFWE Prediction**
Cluster 1	139 voxel [Center: 0/34/-16]	139 voxel [Center: 0/34/-16]
	**TFCE MonteCarlo**	**TFCE Prediction**
Cluster 1	84 voxel [Center: 0/34/-14]	75 voxel [Center: 0/34/-14]

**Cognitive Empathy**		
	**vFWE MonteCarlo**	**vFWE Prediction**
No Clusters		
	**cFWE MonteCarlo**	**cFWE Prediction**
Cluster 1	353 voxel [Center: -2/24/40]	353 voxel [Center: -2/24/40]

Cluster 2	117 voxel [Center: -62/-22/32]	117 voxel [Center: -62/-22/32]
Cluster 3		91 voxel [Center: -40/12/-4]
	**TFCE MonteCarlo**	**TFCE Prediction**
Cluster 1	551 voxel [Center: -2/26/40]	551 voxel [Center: -2/26/40]
Cluster 2	69 voxel [Center: -62/-22/32]	60 voxel [Center: -62/-22/32]

**Affective Empathy**		
	**vFWE MonteCarlo**	**vFWE Prediction**
No Clusters		
	**cFWE MonteCarlo**	**cFWE Prediction**
Cluster 1	107 voxel [Center: -2/16/58]	107 voxel [Center: -2/16/58]
Cluster 2	102 voxel [Center: -49/28/-4]	102 voxel [Center: -49/28/-4]
	**TFCE MonteCarlo**	**TFCE Prediction**
Cluster 1	58 voxel [Center: -49/28/-4]	58 voxel [Center: -49/28/-4]

**Empathy for Emotion**		
	**vFWE MonteCarlo**	**vFWE Prediction**
No Clusters		
	**cFWE MonteCarlo**	**cFWE Prediction**
Cluster 1	385 voxel [Center: 0/-54/30]	385 voxel [Center: 0/-54/30]
Cluster 2	289 voxel [Center: -52/28/-2]	289 voxel [Center: -52/28/-2]
Cluster 3	149 voxel [Center: -56/-60/22]	149 voxel [Center: -56/-60/22]
Cluster 4	147 voxel [Center: -8/54/32]	147 voxel [Center: -8/54/32]
Cluster 5	141 voxel [Center: -2/18/52]	141 voxel [Center: -2/18/52]
Cluster 6	99 voxel [Center: 30/-82/-38]	99 voxel [Center: 30/-82/-38]
	**TFCE MonteCarlo**	**TFCE Prediction**
Cluster 1	375 voxel [Center: 0/-54/30]	375 voxel [Center: 0/-54/30]
Cluster 2	241 voxel [Center: -52/28/-2]	241 voxel [Center: -52/28/-2]
Cluster 3	194 voxel [Center: -8/54/32]	194 voxel [Center: -8/54/32]
Cluster 4	94 voxel [Center: 6/58/22]	119 voxel [Center: 6/58/22]
Cluster 5	64 voxel [Center: -1/18/50]	64 voxel [Center: -1/18/50]
Cluster 6	56 voxel [Center: -56/-60/22]	68 voxel [Center: -56/-60/22]

**Empathy for Pain**		
	**vFWE MonteCarlo**	**vFWE Prediction**
Cluster 1	74 voxel [Center: -2/22/38]	74 voxel [Center: -2/22/38]
	**cFWE MonteCarlo**	**cFWE Prediction**
Cluster 1	488 voxel [Center: -2/22/38]	488 voxel [Center: -2/22/38]
Cluster 2	325 voxel [Center: -60/-24/36]	325 voxel [Center: -60/-24/36]

Cluster 3	195 voxel [Center: 40/12/-4]	195 voxel [Center: 40/12/-4]
Cluster 4	188 voxel [Center: -38/16/-4]	188 voxel [Center: -38/16/-4]
	**TFCE MonteCarlo**	**TFCE Prediction**
Cluster 1	617 voxel [Center: -2/22/38]	617 voxel [Center: -2/22/38]
Cluster 2	476 voxel [Center: -60/-22/34]	476 voxel [Center: -60/-22/34]
Cluster 3	155 voxel [Center: -38/16/-4]	155 voxel [Center: -38/16/-4]
Cluster 4	125 voxel [Center: 40/12/-4]	103 voxel [Center: 40/12/-4]
Cluster 5	32 voxel [Center: -58/10/4]	32 voxel [Center: -58/10/4]

**Time Perception**		
	**vFWE MonteCarlo**	**vFWE Prediction**
Cluster 1	470 voxel [Center: 2/14/52]	494 voxel [Center: 2/14/52]
Cluster 2	240 voxel [Center: -40/18/0]	261 voxel [Center: -40/18/0]
Cluster 3	206 voxel [Center: 38/20/0]	219 voxel [Center: 38/20/0]
Cluster 4	103 voxel [Center: 18/8/4]	109 voxel [Center: 18/8/4]
Cluster 5	72 voxel [Center: 52/16/10]	75 voxel [Center: 52/16/10]
Cluster 6	60 voxel [Center: 52/12/22]	64 voxel [Center: 52/12/22]
	**cFWE MonteCarlo**	**cFWE Prediction**
Cluster 1	1495 voxel [Center: 2/12/54]	1495 voxel [Center: 2/12/54]
Cluster 2	1327 voxel [Center: -40/14/2]	1327 voxel [Center: -40/14/2]
Cluster 3	1326 voxel [Center: 46/16/4]	1326 voxel [Center: 46/16/4]
Cluster 4	546 voxel [Center: 20/6/2]	546 voxel [Center: 20/6/2]
Cluster 5	430 voxel [Center: 48/-40/46]	430 voxel [Center: 48/-40/46]
Cluster 6	325 voxel [Center: 42/40/22]	325 voxel [Center: 42/40/22]
Cluster 7	272 voxel [Center: -32/-64/-32]	272 voxel [Center: -32/-64/-32]
Cluster 8	238 voxel [Center: -42/-48/44]	238 voxel [Center: -42/-48/44]
	**TFCE MonteCarlo**	**TFCE Prediction**
Cluster 1	4404 voxel [Center: 40/16/6]	4404 voxel [Center: 40/16/6]
Cluster 2	2413 voxel [Center: 2/10/54]	2413 voxel [Center: 2/10/54]
Cluster 3	2332 voxel [Center: -42/12/2]	2332 voxel [Center: -42/12/2]
Cluster 4	386 voxel [Center: 48/-40/46]	386 voxel [Center: 48/-40/46]
Cluster 5	365 voxel [Center: 32/4/56]	365 voxel [Center: 32/4/56]
Cluster 6	310 voxel [Center: -42/-46/44]	310 voxel [Center: -42/-46/44]
Cluster 7	130 voxel [Center: -32/-64/-32]	130 voxel [Center: -32/-64/-32]
Cluster 8	115 voxel [Center: 50/10/42]	57 voxel [Center: 50/8/44]

**Late-life Depression**		
	**vFWE MonteCarlo**	**vFWE Prediction**
No Clusters		

	cFWE MonteCarlo	cFWE Prediction
No Clusters		
	**TFCE MonteCarlo**	**TFCE Prediction**
No Clusters		

	**Narcolepsy**	
	**vFWE MonteCarlo**	**vFWE Prediction**
No Clusters		
	**cFWE MonteCarlo**	**cFWE Prediction**
No Clusters		
	**TFCE MonteCarlo**	**TFCE Prediction**
No Clusters		

	**Pain Kogler**	
	**vFWE MonteCarlo**	**vFWE Prediction**
Cluster 1	500 voxel [Center: 40/14/2]	496 voxel [Center: 40/14/2]
Cluster 2	480 voxel [Center: 2/14/38]	471 voxel [Center: 2/14/38]
Cluster 3	330 voxel [Center: 58/-24/22]	329 voxel [Center: 58/-24/22]
Cluster 4	218 voxel [Center: -54/-20/18]	218 voxel [Center: -54/-20/18]
Cluster 5	199 voxel [Center: -36/16/6]	197 voxel [Center: -36/16/4]
Cluster 6	124 voxel [Center: -12/-14/6]	122 voxel [Center: -12/-14/6]
Cluster 7	73 voxel [Center: 44/46/10]	71 voxel [Center: 44/46/10]
Cluster 8	61 voxel [Center: 12/-14/4]	60 voxel [Center: 12/-14/4]
Cluster 9	44 voxel [Center: -41/-2/8]	41 voxel [Center: -42/-2/8]
	**cFWE MonteCarlo**	**cFWE Prediction**
Cluster 1	7400 voxel [Center: 10/-2/8]	7400 voxel [Center: 10/-2/8]
Cluster 2	1913 voxel [Center: 0/14/40]	1913 voxel [Center: 0/14/40]
Cluster 3	659 voxel [Center: 44/46/10]	659 voxel [Center: 44/46/10]
Cluster 4	195 voxel [Center: -36/44/22]	195 voxel [Center: -36/44/22]
Cluster 5	139 voxel [Center: -26/-64/-22]	139 voxel [Center: -26/-64/-22]
Cluster 6		108 voxel [Center: -34/-57/-32]
	**TFCE MonteCarlo**	**TFCE Prediction**
Cluster 1	19012 voxel [Center: 14/-4/8]	19012 voxel [Center: 14/-4/8]
Cluster 2	3511 voxel [Center: 0/14/40]	3511 voxel [Center: 0/14/40]
Cluster 3	114 voxel [Center: 44/-50/52]	142 voxel [Center: 44/-50/51]
Cluster 4	74 voxel [Center: -26/-62/-22]	74 voxel [Center: -26/-62/-22]
Cluster 5	44 voxel [Center: -34/-58/-32]	55 voxel [Center: -34/-58/-32]
Cluster 6	24 voxel [Center: 25/0/-21]	33 voxel [Center: 26/0/-22]

Stress All		
	**vFWE MonteCarlo**	**vFWE Prediction**
Cluster 1	520 voxel [Center: 38/16/0]	528 voxel [Center: 38/16/0]
Cluster 2	438 voxel [Center: 2/10/44]	446 voxel [Center: 2/10/44]
Cluster 3	252 voxel [Center: -36/14/4]	256 voxel [Center: -36/14/4]
Cluster 4	169 voxel [Center: 10/-18/4]	172 voxel [Center: 10/-18/4]
Cluster 5	107 voxel [Center: -16/-12/10]	108 voxel [Center: -16/-12/10]
Cluster 6	95 voxel [Center: 56/-24/24]	95 voxel [Center: 56/-24/24]
Cluster 7	73 voxel [Center: -54/-24/22]	76 voxel [Center: -54/-24/22]
Cluster 8	46 voxel [Center: -42/-18/18]	46 voxel [Center: -42/-18/18]
Cluster 9	32 voxel [Center: 60/6/2]	34 voxel [Center: 60/6/2]
	**cFWE MonteCarlo**	**cFWE Prediction**
Cluster 1	4578 voxel [Center: 10/8/2]	4578 voxel [Center: 10/8/2]
Cluster 2	1416 voxel [Center: 2/12/42]	1416 voxel [Center: 2/12/42]
Cluster 3	836 voxel [Center: 56/-22/22]	836 voxel [Center: 56/-22/22]
Cluster 4	763 voxel [Center: -52/-22/20]	763 voxel [Center: -52/-22/20]
Cluster 5	157 voxel [Center: 38/50/12]	157 voxel [Center: 38/50/12]
	**TFCE MonteCarlo**	**TFCE Prediction**
Cluster 1	13585 voxel [Center: 6/-4/6]	13585 voxel [Center: 6/-4/6]
Cluster 2	2087 voxel [Center: 2/12/40]	2087 voxel [Center: 2/12/40]
Cluster 3	69 voxel [Center: 38/50/12]	69 voxel [Center: 38/50/12]
Cluster 4	41 voxel [Center: -44/-44/42]	41 voxel [Center: -44/-44/42]

Stress Physical		
	**vFWE MonteCarlo**	**vFWE Prediction**
Cluster 1	704 voxel [Center: 40/12/0]	723 voxel [Center: 40/12/0]
Cluster 2	635 voxel [Center: 2/10/42]	652 voxel [Center: 2/10/42]
Cluster 3	390 voxel [Center: -38/12/4]	407 voxel [Center: -38/12/4]
Cluster 4	238 voxel [Center: 10/-16/4]	254 voxel [Center: 10/-16/4]
Cluster 5	165 voxel [Center: -14/-12/10]	167 voxel [Center: -14/-12/10]
Cluster 6	160 voxel [Center: 56/-24/24]	166 voxel [Center: 56/-24/24]
Cluster 7	96 voxel [Center: -54/-24/22]	106 voxel [Center: -54/-24/22]
Cluster 8	67 voxel [Center: -42/-18/18]	71 voxel [Center: -42/-18/18]
Cluster 9	39 voxel [Center: 22/-2/-2]	44 voxel [Center: 22/-2/-2]
Cluster 10	36 voxel [Center: 38/50/12]	40 voxel [Center: 38/49/12]
Cluster 11	26 voxel [Center: -57/0/6]	31 voxel [Center: -56/0/6]
	**cFWE MonteCarlo**	**cFWE Prediction**
Cluster 1	5631 voxel [Center: 6/4/2]	5631 voxel [Center: 6/4/2]
Cluster 2	1654 voxel [Center: 2/12/42]	1654 voxel [Center: 2/12/42]

Cluster 3	878 voxel [Center: -52/-22/20]	878 voxel [Center: -52/-22/20]
Cluster 4	865 voxel [Center: 56/-20/22]	865 voxel [Center: 56/-20/22]
Cluster 5	250 voxel [Center: 40/50/12]	250 voxel [Center: 40/50/12]
	**TFCE MonteCarlo**	**TFCE Prediction**
Cluster 1	14216 voxel [Center: 0/-4/6]	14216 voxel [Center: 0/-4/6]
Cluster 2	2286 voxel [Center: 2/12/42]	2505 voxel [Center: 2/12/42]
Cluster 3	226 voxel [Center: 40/50/12]	226 voxel [Center: 40/50/12]
Cluster 4	106 voxel [Center: 0/-22/30]	140 voxel [Center: 0/-22/30]
Cluster 5	39 voxel [Center: -42/-42/42]	89 voxel [Center: -42/-42/42]
Cluster 6	20 voxel [Center: 42/50/-8]	32 voxel [Center: 42/50/-8]

**Stress Social**		
	**vFWE MonteCarlo**	**vFWE Prediction**
No Clusters		
	**cFWE MonteCarlo**	**cFWE Prediction**
Cluster 1	90 voxel [Center: -12/16/-6]	90 voxel [Center: -12/16/-6]
	**TFCE MonteCarlo**	**TFCE Prediction**
Cluster 1	40 voxel [Center: -12/16/-6]	19 voxel [Center: -12/16/-6]

**Sustained Attention**		
	**vFWE MonteCarlo**	**vFWE Prediction**
Cluster 1	352 voxel [Center: 0/8/48]	389 voxel [Center: 0/8/48]
Cluster 2	221 voxel [Center: 36/18/4]	239 voxel [Center: 36/20/4]
Cluster 3	201 voxel [Center: 48/4/34]	226 voxel [Center: 48/4/34]
Cluster 4	38 voxel [Center: 58/-34/14]	47 voxel [Center: 58/-34/14]
Cluster 5	31 voxel [Center: 6/-12/8]	38 voxel [Center: 6/-13/8]
	**cFWE MonteCarlo**	**cFWE Prediction**
Cluster 1	1243 voxel [Center: 0/10/46]	1243 voxel [Center: 0/10/46]
Cluster 2	924 voxel [Center: 46/4/40]	924 voxel [Center: 46/4/40]
Cluster 3	646 voxel [Center: 38/20/2]	646 voxel [Center: 38/20/2]
Cluster 4	523 voxel [Center: 40/36/24]	523 voxel [Center: 40/36/24]
Cluster 5	438 voxel [Center: 56/-34/16]	438 voxel [Center: 56/-34/16]
Cluster 6	361 voxel [Center: -40/-12/52]	361 voxel [Center: -40/-12/52]
Cluster 7	289 voxel [Center: -48/6/28]	289 voxel [Center: -48/6/28]
Cluster 8	248 voxel [Center: -34/12/4]	248 voxel [Center: -34/12/4]
Cluster 9	199 voxel [Center: 2/-66/-18]	199 voxel [Center: 2/-66/-18]
Cluster 10	196 voxel [Center: -44/-68/-4]	196 voxel [Center: -44/-68/-4]
Cluster 11	196 voxel [Center: 28/-86/2]	196 voxel [Center: 28/-86/2]
Cluster 12	195 voxel [Center: 6/-12/8]	195 voxel [Center: 6/-12/8]

Cluster 13	130 voxel [Center: 36/-60/50]	130 voxel [Center: 36/-60/50]
Cluster 14	130 voxel [Center: -10/-14/8]	130 voxel [Center: -10/-14/8]
Cluster 15	116 voxel [Center: 40/-46/44]	116 voxel [Center: 40/-46/44]
Cluster 16	104 voxel [Center: -32/-58/-26]	104 voxel [Center: -32/-58/-26]
	**TFCE MonteCarlo**	**TFCE Prediction**
Cluster 1	3741 voxel [Center: 40/14/24]	3741 voxel [Center: 40/14/24]
Cluster 2	1659 voxel [Center: 2/10/46]	1659 voxel [Center: 2/10/46]
Cluster 3	1106 voxel [Center: -44/-2/48]	1266 voxel [Center: -44/-2/46]
Cluster 4	514 voxel [Center: 56/-34/16]	590 voxel [Center: 56/-34/16]
Cluster 5	356 voxel [Center: -34/12/4]	424 voxel [Center: -34/12/4]
Cluster 6	327 voxel [Center: 38/-52/48]	327 voxel [Center: 38/-52/48]
Cluster 7	195 voxel [Center: 6/-12/8]	195 voxel [Center: 6/-12/8]
Cluster 8	163 voxel [Center: -44/-68/-4]	196 voxel [Center: -44/-68/-4]
Cluster 9	130 voxel [Center: -10/-14/8]	130 voxel [Center: -10/-14/8]
Cluster 10	113 voxel [Center: 28/-86/2]	113 voxel [Center: 28/-86/2]
Cluster 11	94 voxel [Center: 2/-66/-18]	94 voxel [Center: 2/-66/-18]
Cluster 12	82 voxel [Center: -32/-58/-26]	82 voxel [Center: -32/-58/-26]

	**Depression All**	
	**vFWE MonteCarlo**	**vFWE Prediction**
No Clusters		
	**cFWE MonteCarlo**	**cFWE Prediction**
No Clusters		
	**TFCE MonteCarlo**	**TFCE Prediction**
No Clusters		

	**Depression Activation**	
	**vFWE MonteCarlo**	**vFWE Prediction**
No Clusters		
	**cFWE MonteCarlo**	**cFWE Prediction**
No Clusters		
	**TFCE MonteCarlo**	**TFCE Prediction**
No Clusters		

	**Depression Deactivation**	
	**vFWE MonteCarlo**	**vFWE Prediction**
No Clusters		
	**cFWE MonteCarlo**	**cFWE Prediction**
No Clusters		

	TFCE MonteCarlo	TFCE Prediction
No Clusters		

Depression Cognition		
	vFWE MonteCarlo	vFWE Prediction
No Clusters		
	cFWE MonteCarlo	cFWE Prediction
No Clusters		
	TFCE MonteCarlo	TFCE Prediction
No Clusters		

Depression Emotion		
	vFWE MonteCarlo	vFWE Prediction
No Clusters		
	cFWE MonteCarlo	cFWE Prediction
No Clusters		
	TFCE MonteCarlo	TFCE Prediction
No Clusters		
